# The Vaccine Godmother: Dr. Gagandeep Kang’s Pioneering Journey in Global Health and Vaccine Development

**DOI:** 10.7759/cureus.70184

**Published:** 2024-09-25

**Authors:** Lavanya Balaji, Lavanya Ramanan, Subbalakshmi Easwaran

**Affiliations:** 1 Department of Microbiology, Saveetha Medical College and Hospitals, Saveetha Institute of Medical and Technical Sciences, Saveetha University, Chennai, IND

**Keywords:** gagandeep kang, historical vignette, public and environmental health, rotavirus vaccine, vaccine godmother

## Abstract

Dr. Gagandeep Kang is a distinguished Indian microbiologist and virologist known for her pioneering work in the study of gastrointestinal diseases, diarrheal infections, and vaccine development. This article highlights her career, beginning with her medical education at Christian Medical College (CMC) Vellore, where she embarked on her groundbreaking research in enteric diseases, particularly rotavirus, a major cause of child mortality globally. Over the course of her distinguished career, she has led groundbreaking research on rotavirus, contributing to the development of two WHO-approved vaccines tailored for Indian communities: Rotavac (Bharat Biotech, Hyderabad, India) and Rotasiil (Serum Institute of India, Pune, India). Beyond her research, Dr. Kang has held significant advisory roles on national and global platforms, including WHO’s Global Advisory Committee on Vaccine Safety and the Coalition for Epidemic Preparedness Innovations (CEPI). She has authored over 300 scientific publications and coauthored the bestselling book “Till We Win,” which details India's fight against the COVID-19 pandemic. Her exceptional contributions have earned her numerous accolades, including the Infosys Prize in Life Sciences, the Fellowship of the Royal Society, and the Canada Gairdner Global Health Award. Dr. Kang’s career exemplifies her dedication to advancing public health, vaccine development, and global health initiatives, making her a trailblazer in the field of microbiology and virology.

## Introduction and background

**Figure 1:**
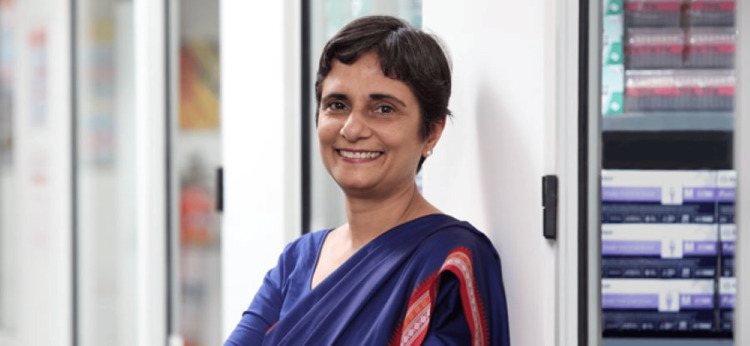
Picture of Dr. Gagandeep Kang Photo credits: Vellore Christian Medical College Foundation [1].

Dr. Gagandeep Cherry Kang (Figure 1) [1], born on November 3, 1962, is an eminent Indian microbiologist and virologist celebrated for her groundbreaking contributions to studying gastrointestinal diseases, diarrheal infections, and disease surveillance. Dr. Kang, originally from Shimla, India, experienced frequent relocations across northern and eastern India during her formative years because of her father's career as a mechanical engineer with the Indian Railways. Her mother, a dedicated English and history educator, instilled in Dr. Kang a deep love for learning, which she nurtured into a fervent passion for science. At the age of 12, she established a home laboratory with her father, initiating experiments in biology, physics, and chemistry, laying the foundation for her future endeavors [2].

Dr. Kang pursued her medical education at Christian Medical College (CMC), Vellore, obtaining her MBBS in 1987, followed by an MD in Microbiology in 1991 and a PhD in 1998. Although she initially considered a career in ophthalmology while at the All-India Institute of Medical Sciences in New Delhi, a familial tremor in her arm redirected her to microbiology, a field where she would go on to make groundbreaking contributions to enteric disease research. Her research journey began earnestly in 1991 when she joined CMC's Wellcome Laboratory as a junior faculty member, focusing on enteric diseases. Dr. Kang's research career has been closely intertwined with her personal life. While serving as a faculty member at CMC Vellore, she pursued her PhD while raising two sons, balancing her demanding career with family responsibilities by living on the hospital campus [3]. CMC appointed her as one of the three core research professors, providing her with full salary support and a primary focus on research. Dr. Kang dedicated her studies to rotavirus, a virus responsible for causing gastroenteritis in children. According to the World Health Organization (WHO), this virus claimed the lives of over 450,000 children in 2008 [4].

In 1998, she expanded her expertise in rotavirus research during a year-long fellowship at the UK Public Health Laboratory Service in London. This was followed by a significant collaboration with Mary K. Estes at Baylor College of Medicine in Houston, USA. This partnership, which spanned several years, proved instrumental in advancing rotavirus research and solidifying Dr. Kang's reputation as a leading figure in global health [5]. Upon her return to India, Dr. Kang initiated extensive field studies to comprehensively understand rotavirus infections in children, reaching a scale not previously undertaken.

## Review

Career beginnings and rotavirus research

**Figure 2:**
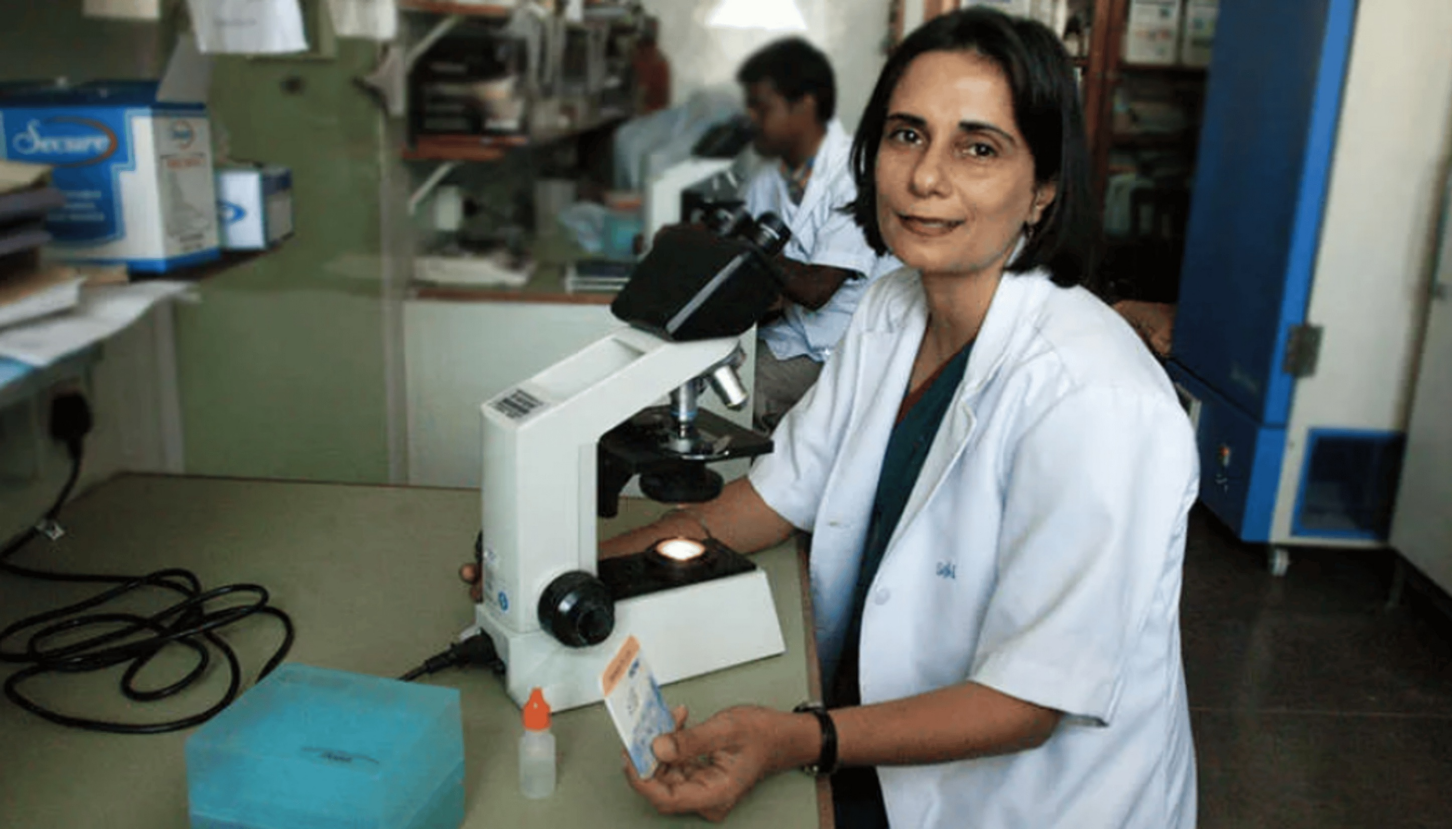
Dr. Gagandeep Kang Picture credit: Getty Images and India Times [4]

Dr. Kang’s research career began in 1991 when she joined CMC Vellore’s Wellcome Laboratory as a junior faculty member (Figure 2). Her focus on enteric diseases, particularly rotavirus infections, soon became a hallmark of her work. During a fellowship in the UK in the late 1990s, she refined her expertise in diarrheal infections and applied these techniques to stool samples collected from India. Supported by her mother, who even bought a fridge to store the samples, Dr. Kang returned to India with 500 samples, which she meticulously tested for rotavirus. Her collaboration with Mary K. Estes at Baylor College of Medicine, one of the world's leading labs for rotavirus research, was particularly influential in her career [5]. The first rotavirus vaccine was withdrawn from the US during this period due to safety concerns. However, Dr. Kang remained committed to developing a vaccine tailored to Indian communities. Her team in Vellore focused on replicating a rotavirus study conducted in Mexico. Although the results in India did not replicate the success seen in Mexico, Dr. Kang and her team spent three years rigorously analyzing the data. Their work led to the groundbreaking conclusion that a vaccine designed specifically for Indian communities could offer about 50% protection, a significant achievement in a region where no protection previously existed [6]. Since the early 1990s, Dr. Kang has dedicated her career to studying diarrheal diseases and improving public health, with a particular focus on rotaviruses and typhoid, which are major causes of illness in children. Her comprehensive approach to vaccine development has involved conducting phase I to III clinical trials and providing essential laboratory support to advance these vaccines [7]. Additionally, Dr. Kang has played a pivotal role in establishing surveillance networks for rotavirus and typhoid in the most affected populations, ensuring that data collection and analysis are robust and relevant. Dr. Kang's efforts have culminated in the development of two WHO-approved vaccines: "Rotavac" (Bharat Biotech, Hyderabad, India) and "Rotasiil" (Serum Institute of India, Pune, India), both of which have made a profound impact on global health. These accomplishments have earned her the title of "Vaccine Godmother" [7-9]. She proudly refers to this accomplishment as "A Vaccine for India, by India, & in India."

Discovering community-centered approaches to address local challenges

In a developing country like India, Dr. Gagandeep Kang has navigated numerous challenges, from limited funding and vaccine hesitancy to difficulties in procuring research materials. With steadfast determination, she has addressed these obstacles while maintaining a clear sense of purpose. During her work monitoring the spread of rotavirus infection in Vellore, she identified a major challenge for impoverished communities: daily wage earners found it difficult to travel to distant hospitals to submit blood and stool samples for their children. In response, she established a clinic within the community, providing free healthcare to children and bringing medical services directly to those in need. This initiative has been running for 18 years, improving countless lives. Dr. Gagandeep’s approach is centered on community involvement, building trust by actively engaging local residents and educating them about clinical trials [10]. Her diverse expertise in medicine and research allows her to lead interdisciplinary projects. She also advocates for essential public health factors such as sanitation, proper nutrition, and clean drinking water, which are crucial for children’s health and disease prevention. Her research, particularly on vaccine trials, plays a significant role in shaping government policies related to vaccination programs.

Literary contributions and advisory roles

Dr. Gagandeep Kang has held several prestigious positions in her distinguished career. She previously served as a professor in the Department of Gastrointestinal Sciences at Christian Medical College (CMC), Vellore, India. From August 2016 to July 2020, she was the Executive Director of the Translational Health Science and Technology Institute (THSTI) in Faridabad, an autonomous institute under the Department of Biotechnology, Ministry of Science and Technology, Government of India [11].

Dr. Kang’s research centers on childhood viral infections with a particular emphasis on the development and evaluation of rotavirus vaccines. Her work extends to the study of other enteric infections that affect children early in life, as well as critical issues surrounding sanitation and water safety. Beyond the lab, Dr. Kang has made her mark in the scientific community, authoring over 300 research papers and serving on the editorial boards of leading journals such as PLoS Neglected Tropical Diseases, International Health, and Current Opinion in Infectious Diseases.

Her influence also reaches the literary world. Dr. Kang co-authored the bestselling book “Till We Win: India's Fight Against the COVID-19 Pandemic” alongside public health expert Dr. Chandrakant Lahariya and AIIMS Director Dr. Randeep Guleria. Published by Penguin Random House India, this collaboration became an essential read during the pandemic, showcasing her ability to communicate complex health issues to a broader audience [12].

Throughout her career, Dr. Kang has been a key figure on numerous review and advisory committees for national and international research funding agencies, especially in the field of vaccines. She has served on India's National Technical Advisory Group on Immunization, the WHO's Global Advisory Committee on Vaccine Safety, and the Immunization and Vaccine Implementation Research Advisory Committee. Since 2015, she has chaired the WHO South-East Asia Region’s Regional Immunization Technical Advisory Group [4].

In addition to her primary roles, Dr. Kang holds honorary appointments as an associate faculty member at the Johns Hopkins University Bloomberg School of Public Health in Baltimore, Maryland, and as an adjunct professor at Tufts University School of Medicine in Boston, Massachusetts. She has been an ex-officio member of a working group on COVID-19 vaccines established by the Strategic Advisory Group of Experts (SAGE) at the WHO, and she played a crucial role in India’s efforts to develop a coronavirus vaccine [13]. She is currently working as Director-EDGE, at the Bill & Melinda Gates Foundation, Seattle, USA.

Global health initiatives and recognitions

Dr. Gagandeep Kang has made a remarkable impact on global health through her influential advisory roles and groundbreaking achievements. Her career is decorated with prestigious honors, including the Dr. P.N. Berry Fellowship (1998-1999), the Lourdu Yedanapalli Award for Excellence in Research (2005), and the Woman Bioscientist of the Year award (2006). Her exceptional contributions were further recognized when she received the Infosys Prize in Life Sciences in 2016, becoming the first Indian woman to be elected to the Fellowship of the American Academy of Microbiology and the only physician-scientist to receive the Infosys Award in Life Sciences and achieve this accolade [14].

Dr. Kang’s dedication to advancing vaccine development was evident during her tenure on the Board of the Coalition for Epidemic Preparedness Innovations (CEPI) from 2018 to 2023, where she played a key role in accelerating vaccines for emerging infectious diseases [15]. In 2019, she was elected a Fellow of the Royal Society, a historic achievement as the first Indian woman to receive this honor in the society's 359-year history. She also made history as the first Indian and first woman to edit Manson's Textbook of Tropical Medicine. Since 2020, Dr. Kang has contributed her expertise to the International Advisory Board at the Global Health Centre of the Graduate Institute of International and Development Studies. Her most recent accolade, the Canada Gairdner Global Health Award in 2024, further underscores her extraordinary impact on global health [16].

## Conclusions

Dr. Gagandeep Kang’s career is a testament to her exceptional contributions to global health, particularly in the fields of enteric diseases and vaccine development. Her pioneering research, combined with her dedication to public health, has had a profound impact on reducing the burden of diarrheal infections worldwide. Dr. Kang’s achievements extend beyond research, encompassing significant advisory roles and literary contributions that have shaped global health policies and practices. As a trailblazer in her field, Dr. Kang continues to inspire and drive progress in global health initiatives, leaving an indelible mark on the world.
